# Competing Effects of Plasticization and Miscibility on the Structure and Dynamics of Natural Rubber: A Comparative Study on Bio and Commercial Plasticizers

**DOI:** 10.1021/acspolymersau.5c00009

**Published:** 2025-04-24

**Authors:** Luca Lenzi, Itziar Mas-Giner, Micaela Degli Esposti, Davide Morselli, Marianella Hernández Santana, Paola Fabbri

**Affiliations:** † Department of Civil, Chemical, Environmental and Materials Engineering (DICAM), 9296 Universitá di Bologna, Via Terracini 28, 40131 Bologna, Italy; ‡ National Interuniversity Consortium of Materials Science and Technology (INSTM), 50121 Firenze, Italy; § Institute of Polymer Science and Technology (ICTP), CSIC, Juan de La Cierva 3, 28006 Madrid, Spain

**Keywords:** natural rubber, epoxidized natural rubber, bioplasticizer, segmental dynamics, sustainable polymer additives

## Abstract

Plasticizers are essential for improving the processability and flexibility of rubber compounds by reducing viscosity, aiding filler dispersion, and softening the rubber matrix. Traditionally, petroleum-based phthalate esters like dioctyl phthalate (DOP) and dibutyl phthalate (DBP) have been widely used for these purposes. However, these plasticizers pose significant challenges, including migration from the rubber over time, which can lower performance and raise environmental and health concerns. This study investigates the competing effects of plasticization and miscibility on the structure and dynamics of natural rubber (NR) and epoxidized natural rubber (ENR) when plasticized with glycerol trilevulinate (GT), a biobased plasticizer, and tris­(2-ethylhexyl) trimellitate (TOTM), a petroleum-derived plasticizer. Results show that GT accelerates vulcanization and reduces reversion risks, promoting faster curing and greater flexibility in the rubber network. In contrast, TOTM delays vulcanization and increases reversion, while forming a more rigid cross-linked network. Structurally, GT promotes longer sulfur bridges and strain-induced crystallization in NR, while TOTM favors the formation of shorter sulfur bonds and a more homogeneous network structure. In terms of miscibility, GT is fully miscible with ENR, improving segmental mobility, but shows partial miscibility in NR, restricting chain dynamics as evidenced by Broadband Dielectric Spectroscopy. These findings highlight GT as a potential sustainable alternative to petroleum-derived commercial plasticizers, offering promising advantages for high-performance, biobased rubber applications.

## Graphical abstract

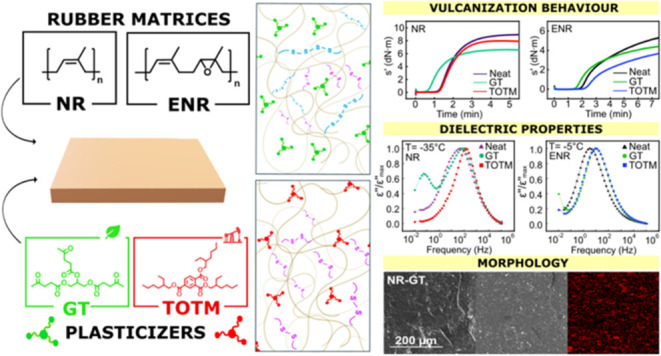

## Introduction

The rubber industry, cornerstone in global manufacturing, plays a crucial role in sectors ranging from automotive to medical devices, where the demand for durable, flexible, and resilient materials is ever-increasing. Natural rubber (NR), predominantly obtained from Hevea brasiliensis, has long been the material of choice due to its unique properties, including high elasticity, tensile strength, and wear resistance.^1^ However, the intrinsic limitations of NR, such as its susceptibility to degradation by oils, chemicals, and environmental factors like ozone and heat, have motivated the development of epoxidized natural rubber (ENR), a chemically modified derivative of NR. By introducing epoxide groups into the polymer backbone, ENR enhances its oil and solvent resistance, broadening its applicability in more demanding environments.^2,3^ ENR has become an essential material in rubber applications requiring improved chemical stability while retaining the flexibility and elasticity that characterizes NR.^3^

Both NR and ENR are typically compounded with additives to further enhance their performance, mainly in processing and mechanical properties.^4^ Among these additives, plasticizers stand out as key components in rubber formulations. Plasticizers improve the workability of rubber compounds by reducing viscosity, facilitating the dispersion of fillers, and softening the rubber matrix, which results in improved processability and flexibility.^5^ The effect of plasticizers on rubber compounds is well documented in the literature,^6^ particularly through models such as the Free Volume Theory and the Lubricity Theory^7^ suggesting that the addition of plasticizers lowers the glass transition temperature (*T*_g_), enhances flow properties, and reduces friction between polymer chains by disrupting intermolecular interactions, leading to more flexible and processable materials. Historically, petroleum derived phthalate esters such as dioctyl phthalate (DOP) and dibutyl phthalate (DBP) have been the most widely used plasticizers, valued for their ability to enhance the flexibility and durability of rubber products.^8^ Despite their effectiveness, these plasticizers are associated with significant drawbacks. They tend to migrate from the rubber matrix over time, which not only compromises the long-term performance of the rubber, but also raises concerns about environmental contamination and human health risks.^9^ For these reasons, the use of these plasticizers in products such as medical devices and children’s toys has led to regulatory restrictions in several countries worldwide. This has resulted in an urgent need for safer, more sustainable alternatives that do not compromise on performance.^10–12^

Recent advances in material science and green chemistry have driven the development of biobased plasticizers, derived from renewable resources.^13–21^ These plasticizers offer several advantages over traditional petroleum-based counterparts, including enhanced biodegradability, lower toxicity, and reduced environmental impact. Notably, biobased plasticizers such as vegetable oil derivatives, fatty acid esters, and glycerol derivatives have shown promise in various polymer systems, including rubbers.^10,22–24^ By leveraging renewable feedstocks, bioplasticizers align with global sustainability goals, such as reducing reliance on fossil fuels and minimizing the ecological footprint of industrial materials.^25^

Within this context, glycerol trilevulinate (GT) has recently emerged as a promising biobased plasticizer characterized by low leachability and high plasticization effect on polymers as poly­(vinyl chloride), polyhydroxyalkanoates and polylactides.^22^ However, GT has never been tested on elastomeric materials. Derived from the esterification of glycerol with levulinic acid, GT is produced through a solvent-free reaction, making it both environmentally^26^ and economically attractive for large-scale production.^22^ Glycerol, a byproduct of the biodiesel industry, and biomass-derived levulinic acid are valorized to obtain a plasticizer that is not only renewable but also highly functional.^23^

The core focus of this study is to evaluate the performance of GT in comparison to a commercial plasticizer, tris­(2-ethylhexyl) trimellitate (TOTM), in both NR and ENR compounds. TOTM is a widely used plasticizer in various applications, particularly where low volatility and high thermal stability are required.^27^ While TOTM provides good thermal stability and resistance to migration,^28^ its structure contains the phthalate moiety, which is associated with potential endocrine-disrupting effects. Furthermore, as a petroleum-derived product, its environmental impact remains a limitation compared to biobased alternatives. The impact of the GT plasticizer on key rubber processing parameters, including viscosity reduction, mechanical properties modification and cross-linking behavior, are compared against the effects of TOTM. Through Fourier-transform infrared (FTIR) spectroscopy, rheometry, and tensile testing, this study provides a detailed examination of how these plasticizers influence performance and long-term stability of rubber products. Additionally, broadband dielectric spectroscopy (BDS) is used to assess the molecular mobility within the rubber matrices, offering insights into the plasticizer’s role in altering the *T*_g_ and other dynamic properties of the rubber.^29^ This research addresses the growing demand for sustainable additives in the rubber industry, offering a potential pathway toward reducing environmental impact, while maintaining or even enhancing the material properties required for industrial applications. The results of this study could contribute to the broader adoption of biobased plasticizers in the rubber industry, aligning with efforts to promote circular economy principles and reduce the carbon footprint of rubber production.

## Experimental Section

### Materials

Natural rubber (NR, SIR10) was supplied from *Indonesian Rubber.* Epoxidized natural rubber (ENR50), with a 50% epoxidation, was purchased from Tun Abdul Razak Research Centre (TARRC) of the Malaysian Rubber Board. GT was synthesized according to the procedure reported elsewhere.^22,23^ The commercial plasticizer tris­(2-ethylhexyl) trimellitate (TOTM, 99%), calcium carbonate (CaCO_3_, ACS reagent, ≥99.0%) and toluene (ACS reagent, ≥ 99.5%) were purchased from Sigma-Aldrich. Sulfur (S, 99%) supplied by Azufrenca, *N*-cyclohexyl-2-benzothiazolsulfenamide (CBS, >98%) from Bayer, zinc oxide (ZnO, 99%) from Minomet C.A., and stearic acid (SA, 99%) from Suministros Químicos were used as components in the vulcanization system. All materials were used as received without further purification.

### Sample Preparation

NR and ENR compounds were prepared following a standard vulcanization formulation.^4^ The formulation included S as vulcanizing agent, CBS as accelerator, and ZnO and SA as activators. CaCO_3_ was added at 30 parts per hundred rubber (phr) as an inert and nonreinforcing filler to facilitate the incorporation of the liquid plasticizer in the two-roll mill.^30^ GT and TOTM plasticizers were each incorporated at a fixed loading of 10 phr (the most typical content for this kind of system) for comparative analysis. Reference samples without plasticizers were also prepared to evaluate the plasticization effect of each additive. The compounds were mixed using a two-roll mill (MGN-300S, Comerio Ercole) at room temperature for 20 min with a friction ratio of 1:1.15. A water circulation system was used during mixing to prevent overheating and prevulcanization. The compounds were prepared following a specific sequence (reported in Table S1) to ensure proper dispersion of each additive. Once mixed, the samples were stored in a freezer for at least 24 h before undergoing further testing. Detailed compositions of the NR and ENR compounds and related sample codes are reported in Table 1.

**Table 1:** Summary of the Formulation and Related Sample Code of the Prepared Natural Rubber (NR) and Epoxidized Natural Rubber (ENR) Compounds

Component^a^	NR-R	NR-GT	NR-TOTM	ENR-R	ENR-GT	ENR-TOTM
NR	100	100	100	-	-	-
ENR	-	-	-	100	100	100
ZnO	5	5	5	5	5	5
SA	1	1	1	1	1	1
CBS	1	1	1	1	1	1
S	2.5	2.5	2.5	2.5	2.5	2.5
GT	-	10	-	-	10	-
TOTM	-	-	10	-	-	10
CaCO_3_	30	30	30	30	30	30

a All values are reported in parts per hundred rubber (phr).

### Rheological Properties

The vulcanization behavior of NR and ENR samples was characterized using a moving die rheometer (MDR 2000, Monsanto), according to ASTM D5289 standard. The samples were positioned between polyester films in the apparatus and tested under isothermal conditions at 160 °C for 120 min at a frequency of 1.7 Hz and an oscillation arc of 0.5°. The measured parameters (collected in Tables S2 and S3) included scorch time (*t*_s2_), indicating the onset of vulcanization, cure time (*t*_90_), representing the time required for the cross-linking reaction to reach 90% of its maximum extent and the difference between maximum torque (*M*_H_) and minimum torque (*M*_L_), reported as Δ*M*.

Reversion (*R*_300_),^31^ which provides an indication of the breakdown of sulfur cross-links in the vulcanized rubber when subjected to prolonged heat or mechanical stress, was calculated using eq 1 in order to evaluate the stability of the rubber network under stress

1 $$R_{300}\;\left(\%\right)=\frac{M_{H}-M_{300s}}{M_{H}}\cdot 100$$

where *M*_300s_ is the difference in torque 300 s after reaching *M*_H^•^_

All compounds were vulcanized by compression molding using a hydraulic press (P 200 P, Collin) at 160 °C and 200 bar. Uncured samples were placed in rectangular molds, positioned between two Teflon sheets to prevent adhesion. Once the compounds reached their respective *t*_90_ values (as detailed in Tables S2 and S3), they were cooled down for 5 min before demolding. Vulcanized sheets, 2 mm thick and 110 mm in length, were used for mechanical testing.

### Cross-Link Density

Swelling measurements were performed, using toluene, on five square specimens (1.5 × 1.5 cm^2^) of each vulcanized compound.^32^ The initial mass of the specimens was first recorded. After immersion in toluene for 72 h, the samples were removed and weighed again. They were subsequently reweighed after allowing the solvent to evaporate. The cross-link density, ρ_cross‑link_ (in moles per volume of rubber, mol·cm^–3^), was determined using the following eq 2

2 $$\rho_{\mathrm{crosslink}}=\frac{\rho_{r}}{2M_{c}}$$

where ρ_r_ is the density of the compound and *M*_c_ the molecular weight between cross-links. The Flory–Rehner equation^33,34^ was applied to calculate the relationship between ρ_r_ and *M*_c_ as follows in eq 3

3 $$\ln\left(1-V_{r}\right)+V_{r}+\chi V_{r}^{2}=-\frac{\rho_{r}}{M_{c}}V_{s}\left(\frac{V_{r}^{1/3}-V_{r}}{V_{r}^{2}}\right)$$

where χ is the Flory–Huggins interaction parameter between the rubbers and the toluene, *V*_s_ is the molar volume of toluene (106.20 cm^3^·mol^–1^ at 25 °C),^35^ and *V*_r_ is the volume fraction of rubber in the compound, which was calculated using the following eq 4

4 $$V_{r}=\frac{\frac{m_{1}}{\rho_{r}}-V_{f}}{\frac{m_{1}}{\rho_{r}}-V_{f}+\left(\frac{m_{2}-m_{3}}{\rho_{s}}\right)}$$

In equation 4, *m*_1_ is the mass of the initial sample before swelling, *m*_2_ is the mass of the swollen sample, *m*_3_ is the mass of the dry sample after solvent evaporation, and ρ_s_ is the density of toluene (0.867 g·cm^–3^).

### Fourier Transform Infrared (FTIR) Spectroscopy

FTIR analysis was conducted to evaluate the difference in the compounds pre and post vulcanization, using a PerkinElmer Spectrum Two spectrometer, fitted with a diamond attenuated total reflection (ATR) crystal. The spectra were collected for each sample over a range of 4000 to 400 cm^–1^, with 16 scans. Data analysis was carried out using Spectrum 10 software (PerkinElmer).

### Thermogravimetric Analysis (TGA)

The thermal stability of the rubber compounds was evaluated using a TGA 2 thermal analyzer (Mettler Toledo). Samples were heated from room temperature to 600 °C under a nitrogen atmosphere, followed by heating up to 800 °C under an oxygen atmosphere, at a rate of 10 °C·min^–1^. Data were processed using TA Universal Analysis software. Onset degradation temperatures are reported in Table S4 in the Supporting Information.

### Mechanical Properties

The mechanical properties of the vulcanized rubber compounds were determined using a universal testing machine (Instron 4204) in accordance with ASTM D412 (2013), with the crosshead speed set at 500 mm·min^–1^ and a 1 kN load cell. Dog bone-shaped samples were tested, and parameters such as modulus at 100, 300, and 500% elongation (*M*_100_, *M*_300_, *M*_500_), tensile strength (σ_break_), and elongation at break (ε_break_) were recorded. Five specimens from each formulation were tested, and the average values are reported alongside their standard deviations in Tables S5 and S6 for NR and ENR compounds, respectively.

### Dielectric Properties

Broadband dielectric spectroscopy (BDS) was performed using a high-resolution dielectric analyzer (α, Novocontrol) to assess the molecular mobility and dynamic properties of the rubber compounds. Films of each sample were placed between two parallel gold electrodes with a diameter of 20 mm. Frequency sweeps were conducted over a range of 10^–1^ to 10^6^ Hz, covering a temperature range from −100 to 100 °C in 5 °C increments.

### Thermal Properties

The thermal properties of the compounds were analyzed using Differential Scanning Calorimetry (DSC, Q10, TA Instruments), equipped with a Discovery Refrigerated Cooling System (RCS90, TA Instruments) under a nitrogen atmosphere (purge flow: 20 mL·min^–1^). Approximately 4 mg of each sample was heated from −90 to 180 °C at a rate of 20 °C·min^–1^ to ensure clear detection of the *T*_g_, reported in Table S4. The resulting thermograms were processed with Universal Analysis 2000 (version 4.5A).

### Morphology and Filler Distribution

Field emission scanning electron microscopy (FE-SEM, Mira3, Tescan) was employed to observe the morphology of the compounds and the filler distribution within the rubber matrices. Cross sections of the rubber samples were prepared by cryo-fracturing in liquid nitrogen. Each sample was mounted on an aluminum stub using a conductive copper tape and coated with approximately 10 nm of gold via the electrodeposition method. Cross-sectional images were obtained at an accelerating voltage of 15 kV using both secondary and backscattered electron detectors. Additionally, elemental distribution, specifically calcium, was mapped through Energy Dispersive X-ray Spectroscopy (EDS).

## Results and Discussion

### Influence of Plasticizers on the Vulcanization Kinetics and Cross-Linking Behavior

The effects of GT and TOTM (which syntheses and structures are reported in Figure 1) on the vulcanization kinetics of NR and ENR were evaluated with both plasticizers incorporated at the fixed loading of 10 phr. CaCO_3_ was added at 30 phr as an inert, non-reinforcing filler^30^ to avoid affecting the properties of the rubber matrix, allowing for the isolation of the effect of each plasticizer on the rubber matrices.

**Figure 1:**
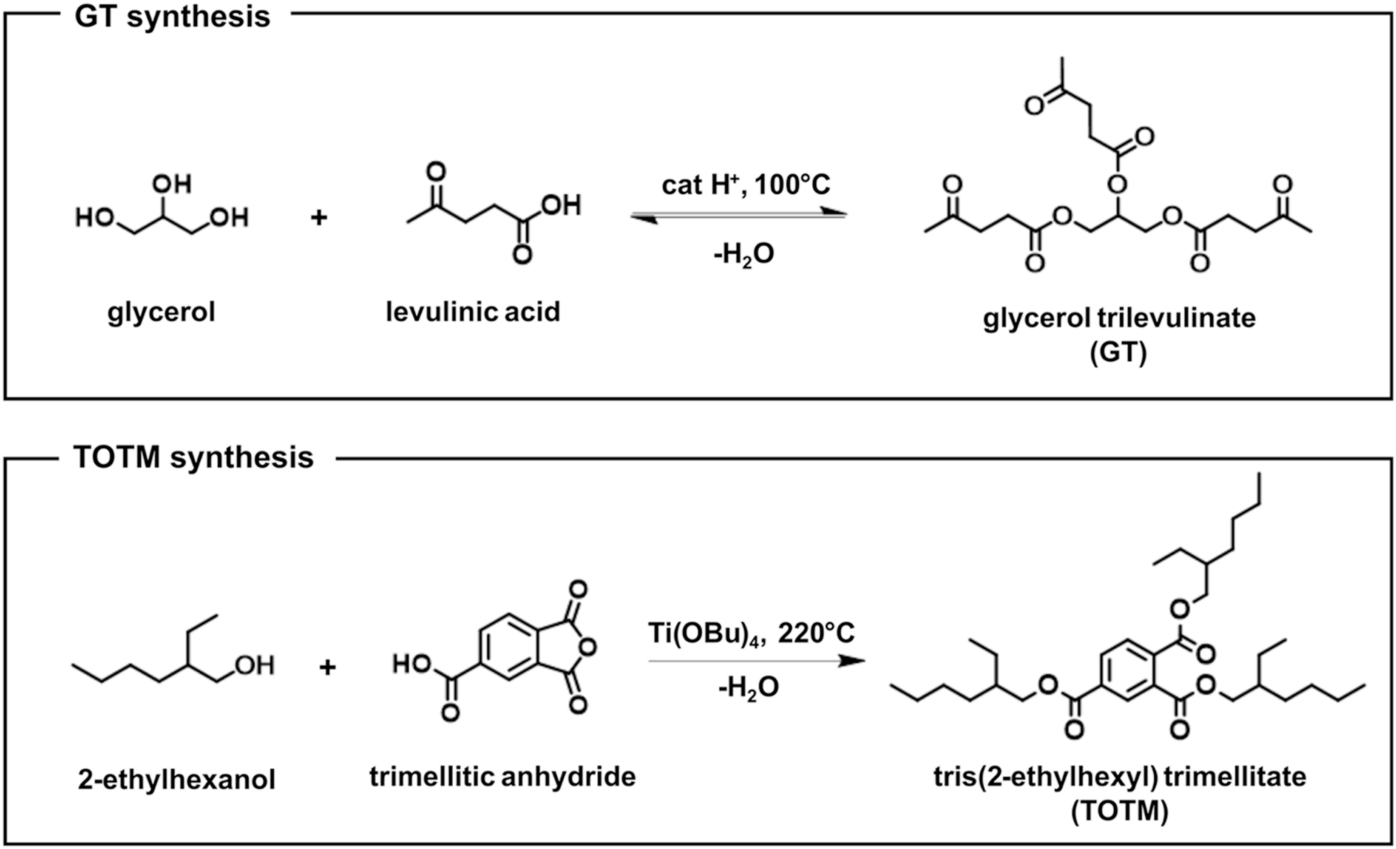
Reaction schemes for the synthesis of both glycerol trilevulinate (GT)^21^ and tris­(2-ethylhexyl) trimellitate (TOTM)^36^ plasticizers.

Monitoring the elastic component of the torque (s′) through time offered key insights into the vulcanization kinetics and cross-linking behavior of the rubber compounds. In particular, the *t*_S2_, *t*_90_, *M*_L_ and *M*_H_ were measured to assess the optimum parameters of vulcanization and the overall effect of the plasticizers on the compounds. These parameters, along with the cross-link density and reversion *R*_300_ are summarized in Tables S2 and S3.

Plasticizers play a complex role influencing the curing behavior of rubber compounds, as shown by the MDR curves in Figure 2A,2B. Novakov et al.^37^ reported how plasticizers generally slowed down the vulcanization process, with the cure rate decreasing as the plasticizer content increased. This reduction was attributed to the formation of kinetically unfavorable associations between the plasticizer molecules and the polysulfide oligomers, which hindered the cross-linking reactions. This effect can lead to a delayed onset of vulcanization, reflected by an increase of *t*_S2_ and *t*_90_ and consequently a decrease of the productivity of the vulcanization process.^10^

**Figure 2:**
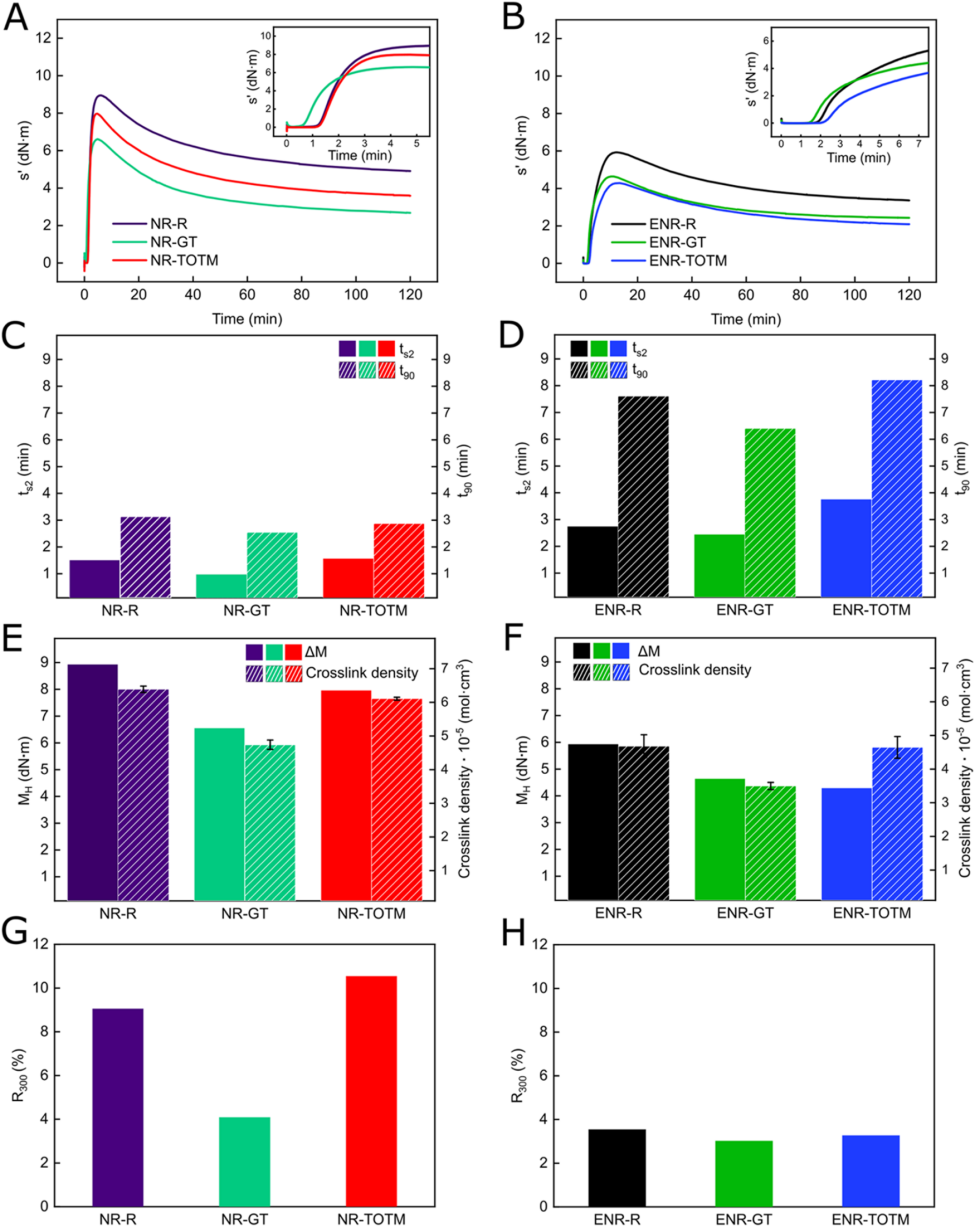
Elastic component of the torque (s') as a function of vulcanization time of (A) NR compounds and (B) ENR compounds. Insets in (A, B) represent the magnified view of the early stages of vulcanization for NR and ENR, respectively. Comparison of *t*_s2_ and *t*_90_ of (C) NR compounds and (D) ENR compounds. M_H_ and the cross-link density of (E) NR samples and (F) ENR samples. *R*_300_ of (G) NR compounds and (H) ENR compounds.

Surprisingly, as visible in the insets of Figure 2A,B, the presence of GT accelerated the onset of the vulcanization process, leading to an earlier initiation of cross-linking. Specifically, as reported in Figure 2C,2D, the inclusion of GT in both NR and ENR reduced *t*_S2_ compared to the reference samples. In NR compounds, GT shortened *t*_S2_ in ∼35% (from 1.51 to 0.97 min), indicating its ability to facilitate early cross-linking interactions. On the contrary, TOTM slightly increased the scorch time (∼5%), suggesting that TOTM may interfere with the initial vulcanization process, delaying cross-linking onset. A similar trend was observed in the ENR samples, where GT reduced *t*_S2_ in ∼10% (from 2.75 min for ENR-R to 2.45 min for ENR-GT), while TOTM further delayed the scorch time to 3.76 min (∼37% more compared to ENR-R). It is noteworthy that the *t*_S2_ values are consistently shorter for the nonpolar compounds (NR-based) compared to their polar counterparts (ENR-based). The strong polar–polar interactions within the ENR likely increased the viscosity, resulting in longer *t*_s2_. This behavior is in accordance with the work of Mensah et al.,^38^ which reported that the initial delay in curing (*t*_s2_) of rubber compounds can be linked to a higher viscosity, slowing the onset of the cross-linking reaction.

The *t*_90_ follows a similar pattern to the scorch time. In NR-based compounds (Figure 2C), GT reduced t_90_ in ∼20% (from 3.14 min for NR-R to 2.54 min for NR-GT), indicating that GT accelerates the overall vulcanization process. TOTM also causes a slight decrease of *t*_90_ (∼10%) with NR-TOTM exhibiting a *t*_90_ of 2.87 min, showing a moderate fastening of the cross-linking reaction. A similar trend is observed in ENR-based compounds (Figure 2D) where GT reduces the *t*_90_ from 7.61 to 6.40 min (∼15% of reduction), while TOTM increases it to 8.21 min (slowing down the process of ∼8%). These results indicate that GT not only accelerates the onset of vulcanization but also promotes faster cross-linking overall, while TOTM appears to hinder the process to some extent, irrespective of the nature of the matrix (NR or ENR). This difference in the vulcanization kinetics could be attributed to the combination of the mechanism by which the plasticizers interact with the vulcanizing agents and the changes in molecular mobility due to the presence of GT and TOTM. In fact, plasticizers like GT can influence the interactions between key vulcanization ingredients such as ZnO, SA, and CBS.^12^ During vulcanization, ZnO and SA react to form zinc stearate,^39^ which plays a critical role in activating the curing system, particularly the activation of accelerators like CBS.^40^

Since plasticizers affect the viscosity of the rubber, they also have a significant impact on the curing behavior of compounds. Plasticizers typically soften the material, reducing *M*_H_ and consequently Δ*M*. As shown in Figure 2E, the incorporation of GT in NR-based compounds reduced *M*_H_, indication of the increased flexibility of the rubber matrix. TOTM-plasticized samples, on the other hand, shows a less pronounced reduction. Similarly, a decrease in *M*_H_ is observed in ENR-based compounds (Figure 2F) with both plasticizers. To better understand the possible action of these additives in the vulcanization process, the cross-link density (strongly related with *M*_H_) was calculated from swelling measurements (eq 2). The addition of GT to both rubber compounds reduced the cross-link density of the rubbers, especially for the NR system (Figure 2E). Meanwhile, the effect of incorporating TOTM was less appreciable in accordance with the *M*_H_ values previously reported. Additionally, all the nonpolar compounds (NR-based) showed higher *M*_H_ values, as well as cross-link density, compared to ENR-based compounds (Figure 2F). This difference is likely due to the stronger polar–polar interactions within the ENR matrix, which may hinder the cross-linking process and reduce the overall network rigidity.^38^

After reaching *M*_H_, NR compounds vulcanized with sulfur often exhibit a drop in torque, a phenomenon known as reversion.^28^ This occurs because continuous shearing during rheological testing can break sulfur cross-link bridges, leading to a reduction in mechanical properties.^41^ Minimizing reversion is crucial for maintaining the long-term mechanical stability of rubber compounds. In this study, reversion was evaluated using the *R*_300_ parameter (calculated with eq 1). As shown in Figure 2G, GT proved highly effective at reducing reversion in NR compounds. The NR reference sample exhibited a *R*_300_ value of ∼9%, while GT significantly lowered this to ∼4%. In contrast, the NR-TOTM sample showed a higher *R*_300_ value of almost 11%, indicating that TOTM is less effective at stabilizing the rubber network under stress. Similar trends were observed in the ENR compounds (Figure 2H), although the effect of both plasticizers was less significant. The improved stability with time of the two rubber matrices with GT is likely due to the plasticizer′s ability to reduce internal friction between rubber macromolecules, resulting in reduced stress applied to the S-bridges of the cross-linking network and, consequently, improving long-term performance of the materials.^31^

### Effect of Plasticizers on the Structure of Rubber Networks

FTIR analyses were conducted to monitor the chemical interactions between the plasticizers and the rubber chains, specifically to determine whether the plasticizers chemically react during the vulcanization process or degrade under the applied thermo-mechanical conditions. As reported in previous studies,^22,23^ GT exhibits a characteristic double peak around 1700 cm^–1^ (Figure 3A). In particular, the left peak at 1733 cm^–1^ corresponds to the stretching vibration of the ester groups in the structure of the plasticizer, while the right peak at 1713 cm^–1^ is attributed to the stretching of the carbonyl group within the ketone moieties. A similar ester bond stretching vibration at 1733 cm^–1^ is also observed in the FTIR spectra of TOTM (Figure 3A). After the vulcanization process, both NR and ENR still displayed the characteristic peaks of the two plasticizers, indicating that the chemical structure of GT and TOTM remained unaltered throughout the process (Figure 3B,C). The presence of these peaks after vulcanization also suggests that neither GT or TOTM react with S during the vulcanization process, and they do not undergo thermo-mechanical degradation. Therefore, the variations of the vulcanization parameters cannot be attributed to undesired chemical reactions between S and the plasticizers. Instead, the distinct effects of GT and TOTM on the formation of sulfur cross-links can be associated with their influence on S solubility and the overall vulcanization mechanism. As suggested by Markov et al.,^42^ plasticizers may impact vulcanization through system dilution. While the formation of sulfur radicals is primarily governed by the vulcanization accelerator system rather than direct solubility effects, solubility still plays a crucial role in determining how sulfur is dispersed within the matrix and its availability for cross-linking.^43^ GT, with its polar nature, likely decreases S solubility within the rubber matrix,^44^ supporting S radicals to form longer S cross-linking bonds. Conversely, TOTM, which contains an aromatic/aliphatic structure, may enhance the solubility of S, facilitating the formation of S radicals and reducing the proportion of polysulfide bonds.^44^ Moreover, despite employing a typical CBS/S ratio of 0.4, which generally promotes polysulfide formation, the presence of TOTM appeared to modulate sulfur solubility and reactivity, thereby increasing the amount of shorter sulfur bonds.

**Figure 3:**
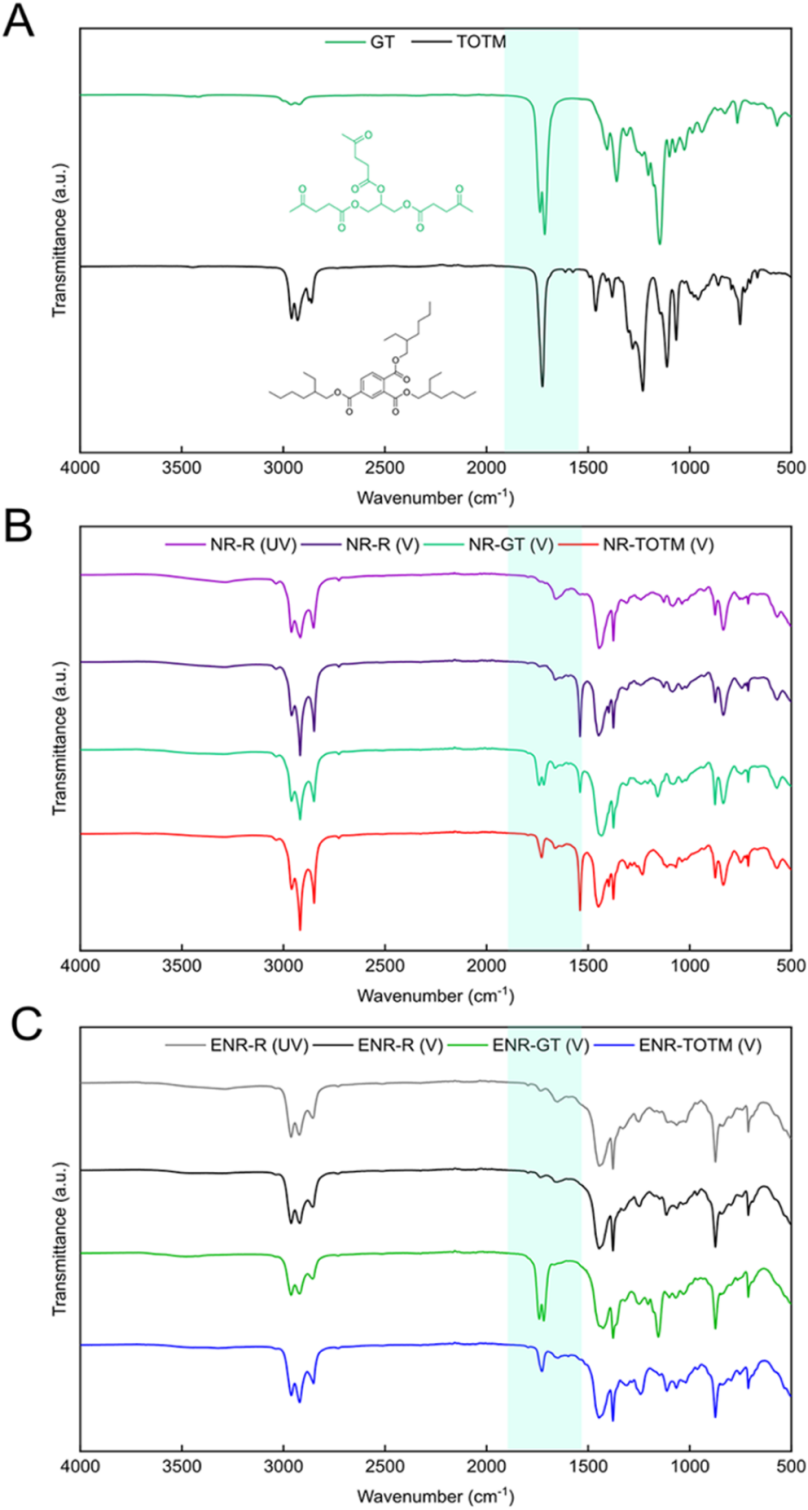
(A) FTIR spectra of GT and TOTM plasticizers; FTIR spectra of the (B) NR compounds and (C) ENR compounds. “(UV)” and “(V)” in the sample codes indicate whether the sample is unvulcanized or vulcanized, respectively.

This hypothesis is reinforced by the cross-link density values, which show that GT compounds are less cross-linked compared to the neat rubbers and TOTM-containing compounds. The presence of shorter S bonds in the latter, resulted in a more densely cross-linked network. Additionally, the lower values of *M*_H_ of NR-GT and ENR-GT compared to the references align with the premise that GT promotes the formation of longer S bridges, which lead to a less cross-linked network. By reducing the viscosity of the system, GT also preserves the integrity of longer S bonds under prolongated thermo-mechanical stress, as demonstrated by the *R*_300_ values (Figure 2G).

The potential presence of longer sulfur bonds was further supported by the results of the TGA investigations (results summarized in Table S4). For NR compounds (Figure S1A), the onset degradation temperature (*T*_d_) of the GT-plasticized rubber (226 °C) was lower than that of the reference compound (262 °C) and the TOTM-plasticized one (244 °C). Similarly, for ENR compounds (Figure S1B), a more pronounced difference in *T*_d_ was observed. The reference ENR compound degraded at a significantly higher temperature (300 °C) compared to ENR-GT (221 °C) and ENR-TOTM (253 °C). This behavior aligns with the well-established understanding that longer sulfur bonds, while promoting flexibility, result in lower thermal stability due to their tendency to break down more readily at elevated temperatures.^45^

The cross-linked network also affected the mechanical properties of the vulcanized rubber compounds, with results summarized in Tables S5 and S6 for ENR and NR samples, respectively. Tensile tests revealed significant differences in the mechanical properties of the rubber compounds, which can be linked to the influence of the plasticizers on the sulfur cross-linked structure. As shown in Figure 4A,4B, both GT and TOTM exhibited a clear plasticizing effect on the mechanical properties of the rubber matrices. The moduli at different elongations (*M*_100_, *M*_300_, and *M*_500_) are typical measures of rubber stiffness. In NR samples (Figure 4C), a reduction in these parameters was observed for both plasticizers, indicating that the rubber reduced its stiffness. A similar trend was seen in ENR samples (Figure 4D), where both plasticizers led to a reduction in *M*_100_, *M*_300_, and *M*_500_, further confirming the plasticizing effect. Regardless the matrix, compounds with GT showed slightly lower modulus at every deformation compared to the compounds with TOTM. This is ascribed to the presence of longer S bonds that lead to increased chain mobility and consequent reduced stiffness. In contrast, TOTM-plasticized rubbers exhibited higher moduli with respect to GT-plasticized compounds, due to the formation of shorter S bridges, resulting in a more rigid network in good agreement with the M_H_ and cross-link density results.

**Figure 4:**
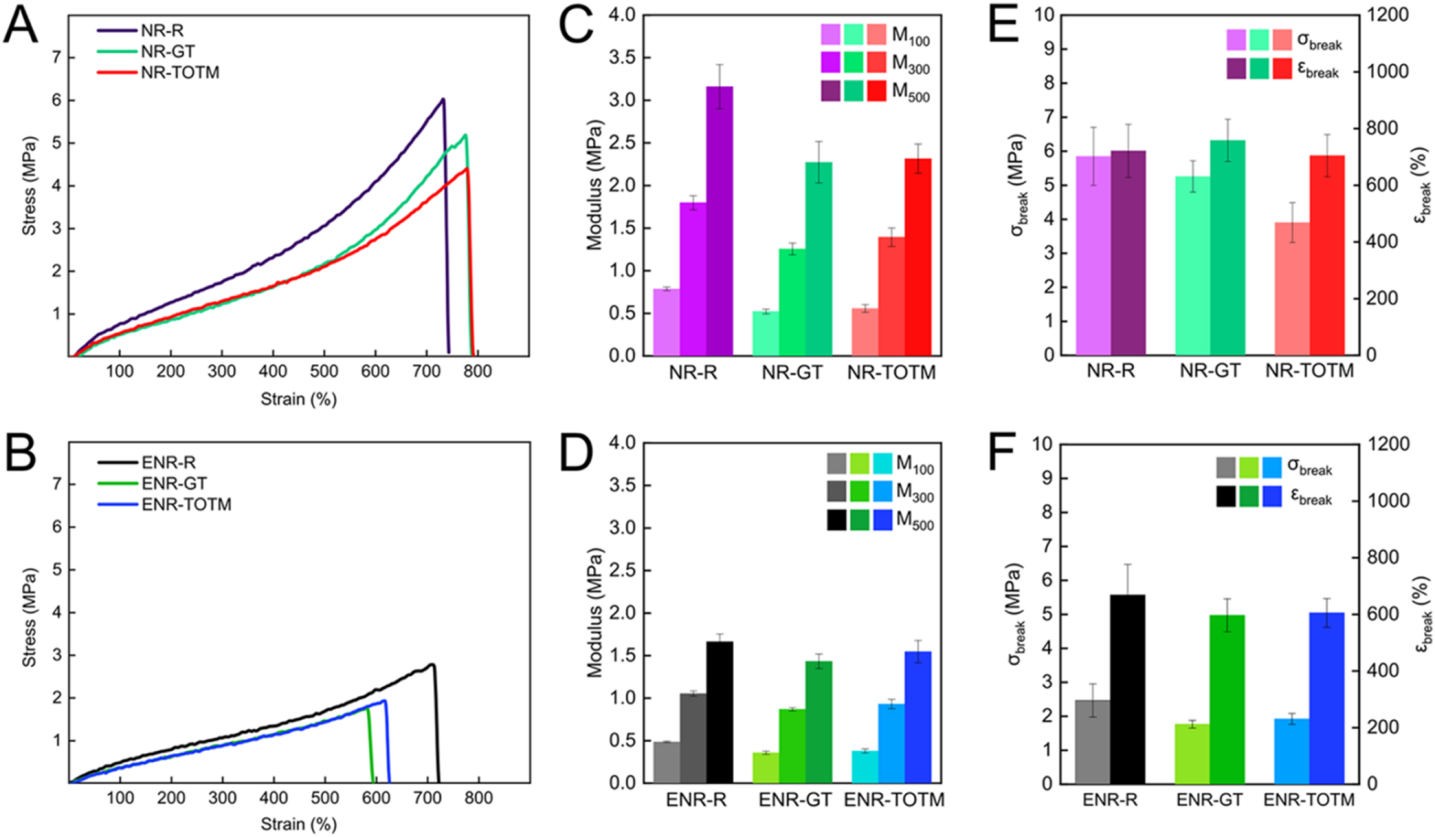
Representative stress–strain curves of (A) NR compounds and (B) ENR compounds. Values of elastic modulus of (C) NR compounds and (D) ENR compounds evaluated at different elongations. Stress and strain at break (σ_break_ and ε_break_ respectively) of (E) NR compounds and (F) ENR compounds.

In terms of the strength at the breaking point (σ_break_), the first outcome is the better performance of all NR-based compounds compared to ENR-based compounds. As expected, the overall higher tensile strength of the NR compounds versus their ENR-based peers is due to the well-known strain induced crystallization (SIC) property of NR. When NR is stretched, the polymer chains are drawn in the direction of the applied stress. It is thought that strain-induced crystallites are formed, acting as additional physical cross-linking points. The crystalline regions provide NR a self-reinforcement character, which is known as the main factor responsible for the toughness of the material.^46^

The mechanical performance of NR-based compounds with GT, which is nearly equivalent to that of neat NR, can be attributed to the enhanced ability of GT to promote SIC. This occurs because GT allows for greater chain mobility due to reduced cross-link density, enabling the formation of additional crystallization points under stress, which act as physical reinforcements. In contrast, TOTM-plasticized compounds exhibit less effective strain-induced crystallization, resulting in lower σ_break_ values (Figure 4E). The effect of SIC is less pronounced in ENR compounds (Figure 4F). This is partially due to the influence of epoxidation, as higher epoxidation levels hinder SIC by restricting chain mobility and disrupting crystallizable sequences.^47^ Additionally, SIC is strongly influenced by cross-link density, with low cross-link densities reducing the ability of polymer chains to orient and crystallize under strain.^48^ Since ENR in our study exhibited both lower cross-link density and modified molecular structure due to epoxidation, the suppression of SIC was expected. Furthermore, the presence of the plasticizer free molecules can also lead to a reduced SIC in rubber compounds, as previously reported.^49^ This trend is consistent with our findings shown in Figure 4F, where SIC effects in ENR appear significantly less pronounced than in NR. The elongation at break (ε_break_) showed different trends among the plasticized compounds. Specifically, for NR-compounds no significant changes were achieved, whereas ENR-compounds exhibited a slight reduction in ε_break_ upon plasticization (Figure 4E,F).

### Understanding the Effect of Plasticizers on the Dynamics of Rubber Networks

Broadband dielectric spectroscopy (BDS) analysis was carried out to gain deeper insight into the molecular dynamics and changes within NR and ENR during plasticization with GT and TOTM. This spectroscopic technique provides valuable information about the multilevel molecular mobility of a rubber compound by measuring its dynamic response to temperature in a wide range of frequencies. The analysis of molecular dipole fluctuations within the system, which are correlated with the mobility of molecular groups, segments, or entire polymer chains, manifests as distinct relaxation processes. In rubber compounds, BDS can uncover crucial transitions, including the α-relaxation, at which large-scale cooperative segmental motions of rubber chains occur.^50^ BDS is also able to detect β-relaxations, which correspond to the local motion of side groups or short chain sections, occurring below the *T*_g_ and often associated with smaller, localized movements within the rubber matrix.^51^

BDS is especially useful as it can show how plasticizers influence the molecular motions of the rubber chains. It detects shifts in relaxation times and changes in dipole behavior, providing insights into the interactions between rubber chains and plasticizers and how these additives influence the material’s flexibility and processability.^29^ Figure 5A,B show the normalized dielectric loss (ε″/ε″_max_) spectra of the NR-based and ENR-based compounds over a wide frequency range and at selected temperatures (−35 and −5 °C, respectively). These temperatures were specifically selected because a relatively broad and asymmetric peak was detected, well-centered and clearly resolved within the frequency window. These maxima can be attributed to the α-relaxation, which is related to the glass transition of the rubbers. One can notice that the inclusion of GT and TOTM in both rubbers shifted the α-relaxation peak to higher frequencies (less restricted dynamics), indicating that the segmental motions of the rubber chains were favored by the presence of both plasticizers. These results are consistent with the previously discussed cross-link density values, where the plasticized compounds exhibited lower cross-linking degree (Figure 2E,2F). This indicates that the inherent constraints on segmental motions imposed by the cross-links are diminished in the presence of plasticizers. Interestingly, NR-GT displayed an additional peak at lower frequencies, which might correspond to the *T*_g_ of GT itself (Figure 5A). In fact, previous studies have reported a *T*_g_ for GT around −49 °C, which is slightly higher than the typical *T*_g_ of NR (approx. −55 °C).^22^ The presence of this secondary peak suggests that GT is not fully miscible with NR’s aliphatic structure, resulting in phase separation, and thus to a second transition, as similarly reported for SSBR/BR blends with coconut oil as plasticizer.^12^ In fact, as reported by van Elburg et al.,^12^ the presence of a shoulder in DMA curves, characterization technique that can be considered comparable or relatable to BDS in detecting phase separation, is indicative of poor compatibility between the plasticizer and polymeric matrix. On the contrary, the ENR-GT compound (Figure 5B) does not exhibit this secondary transition peak. This is likely due to the higher compatibility between GT and ENR, as the polar structure of ENR is more chemically similar to the polar-rich nature of GT, allowing for better miscibility within the matrix.

**Figure 5:**
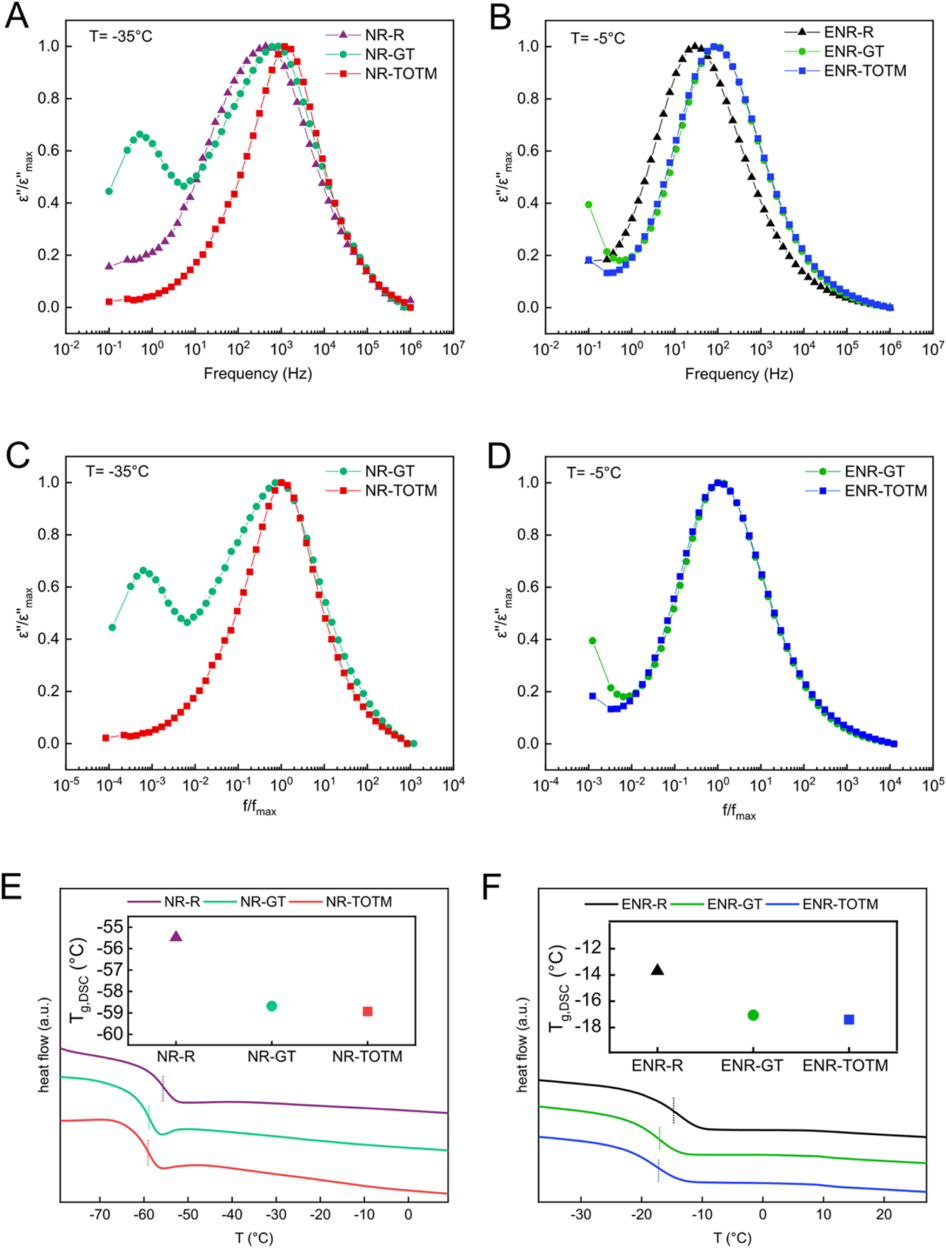
(A) Normalized dielectric loss (ε″/ε″max) vs frequency at −35 °C, showing the α-relaxation region for NR compounds; (B) Normalized dielectric loss (ε″/ε″max) vs frequency at −5 °C, showing the α-relaxation region for ENR compounds; (C) Normalized dielectric loss (ε″/ε″max) vs normalized frequency (*f*/*f*_max_) at −35 °C for NR compounds; (D) Normalized dielectric loss (ε″/ε″max) vs normalized frequency (*f*/*f*_max_) at −5 °C for ENR compounds. DSC thermograms of (E) NR formulations and (F) ENR formulations (insets display the *T*_g,DSC_ for each sample). The dashed lines on the thermograms indicate the heat flow jumps that were used to extrapolate the *T*_g,DSC_ values.

It was initially expected that the maximum loss of the NR-GT compound would appear at higher frequencies (less restricted dynamics) compared to the NR-TOTM compound, due to the lower cross-link density, as previously discussed. However, the results show the opposite. A plausible explanation for this behavior could be the competition between the plasticizing effect, which increases the mobility of adjacent rubber chains, and the partial miscibility of GT in the NR matrix. The latter appears to be the dominant factor, contributing to more constrained movements compared to those observed in the NR-TOTM compound.

A clearer understanding of the plasticizer effect can be extracted from the analysis of the shape (symmetry and width) of the relaxation spectra (Figure 5C,D). One can notice significant differences in the spectra shape by plotting the normalized dielectric loss as a function of the normalized frequency (*f*/*f*_max_). According to the phenomenological model proposed by Schönhals and Schlosser,^52^ the shape of the normalized dielectric loss peak is influenced by the polymer behavior at both low and high frequencies, governed by inter- and intramolecular interactions, respectively. Variations on the low-frequency side may reflect changes in the dynamics of the main chain segments, influenced by the plasticizer contribution.^53^ As reported in Figure 5C, NR in the presence of TOTM displayed a narrower and more symmetrical loss peak, indicating a more homogeneous structure formed by chains with similar mobility, likely due to the presence of short S bonds, as previously discussed. In contrast, the broader and less symmetrical loss peak observed for NR-GT suggests that chain segments have differing dynamics, indicating a higher degree of structural heterogeneity (Figure 5C). This implies regions with distinct mobilities, consistent with the hypothesis of a network composed of longer S bonds. As shown in Figure 5D, the ENR compounds plasticized with both GT and TOTM exhibited a symmetrical loss peak, suggesting that each plasticizer contributed to forming a homogeneously cross-linked network structure within the rubber matrix. This symmetry reflects a balanced distribution of chain mobility across the network, supporting the formation of a consistent structural organization in both systems. These observations align well with the cross-link density values of the ENR compounds (Figure 2F), where similar levels of cross-linking were achieved with GT and TOTM, confirming the comparable effectiveness of each plasticizer in promoting uniformity in the rubber matrix.

To gain a deeper understanding of the influence of plasticizer solubility on sulfur dispersion in rubber matrices, solubility parameters were calculated using the Hoftyzer–Van Krevelen method.^54^ Solubility parameters provide a measure of the cohesive energy density within materials, helping to estimate the compatibility between different components. According to this theory, substances with similar solubility parameters tend to exhibit better miscibility due to more favorable intermolecular interactions. The total solubility parameter (δ) is defined by eq 5

5 $$\delta =\sqrt{\delta_{d}^{2}+\delta_{p}^{2}+\delta_{h}^{2}}$$

where δ_d_, δ_p_, and δ_h_ represent the dispersion, polar, and hydrogen bonding contributions, respectively. The calculated solubility parameters for NR, ENR, GT, TOTM, and sulfur are reported in Table 2.

**Table 2:** Solubility Parameters (δ) of PLA, GT, and DINCH Calculated Using the Hoftyzer–Van Krevelen Method^54^,^a^

Material	δ_d_ (MJ/m^3^)^1/2^	δ_p_ (MJ/m^3^)^1/2^	δ_h_ (MJ/m^3^)^1/2^	δ (MJ/m^3^)^1/2^
NR	17.70	0.00	0.00	17.70
ENR	13.90	2.42	4.26	14.74
GT	15.20	6.33	8.61	18.94
TOTM	16.75	2.86	6.17	18.07
Sulfur	33.10	0.00	0.00	33.10

a The δ_d_, δ_p_, and δ_h_ contributions were used to determine the solubility parameters for each component.

To assess the compatibility between sulfur, plasticizers and the rubber matrices, the difference in solubility parameters (Δδ) was determined using eq 6

6 $$\Delta \delta =\sqrt{{\left(\delta_{d,\mathrm{pol}}-\delta_{d,\mathrm{add}}\right)}^{2}+{\left(\delta_{p,\mathrm{pol}}-\delta_{p,\mathrm{add}}\right)}^{2}+{\left(\delta_{h,\mathrm{pol}}-\delta_{h,\mathrm{add}}\right)}^{2}}$$

where δ_pol_ and δ_add_ refer to the solubility parameters of the polymer and additive, respectively. A commonly accepted criteria suggests that a Δδ value below 5 MPa^1/2^ indicates good compatibility, while higher values suggest reduced miscibility.

The results showed that TOTM exhibited better compatibility with both NR (Δδ = 6.86 MPa^1/2^) and sulfur (Δδ = 17.71 MPa^1/2^), suggesting that TOTM promoted a more homogeneous dispersion of sulfur within the rubber matrix. This improved dispersion may facilitate a more uniform distribution of reactive sulfur species, influencing the cross-linking process. A well-dispersed sulfur phase enhances the formation of sulfur radicals, which are crucial for cross-linking, and may favor the prevalence of shorter sulfur cross-links (monosulfides and disulfides) over polysulfidic structures. Despite employing a typical CBS/S ratio of 0.4, which generally promotes polysulfide formation, the presence of TOTM appeared to modulate sulfur solubility and reactivity, thereby increasing the amount of shorter sulfur bonds. Conversely, GT, due to its higher polarity, demonstrates lower compatibility with NR (Δδ = 11.58 MPa^1/2^) and sulfur (Δδ = 21.17 MPa^1/2^), potentially leading to a more localized distribution of sulfur within the rubber phase. This nonuniform dispersion may result in regions of higher sulfur concentration, which can influence the kinetics of radical formation and promote the formation of longer polysulfidic linkages. However, ENR, with its increased polarity due to epoxidation, exhibits a stronger interaction with GT (Δδ = 5.68 MPa^1/2^), suggesting a better dispersion and interaction of GT within the ENR matrix compared to NR.

DSC investigations were carried out to confirm the plasticization effect of GT and TOTM on NR and ENR (results in Table S4). As shown in Figure 5E, both GT and TOTM effectively plasticized the NR compounds, leading to a noticeable reduction in *T*_g_. Specifically, the *T*_g_ decreased from −55 °C in the unplasticized NR to −59 °C when either GT or TOTM was incorporated, demonstrating comparable plasticizing efficiency for both additives. Unexpectedly, despite BDS analysis suggesting phase separation in NR-GT (Figure 5A), only a single transition was observed in the thermograms of NR compounded with GT (Figure 5E). This was likely because the *T*_g_ values of neat NR (−55 °C) and neat GT (−49 °C) were so close that individual transitions could not be distinguished. ENR compounds also exhibited a *T*_g_ reduction upon plasticization with these additives. As reported in Figure 5F, the *T*_g_ of ENR-R decreased from −14 to −17 °C, indicating that both plasticizers promoted increased segmental mobility of the rubber chains.

SEM imaging was performed to examine the morphology of the plasticized compounds, focusing on the potential rubber matrix/plasticizer phase separation. Secondary electron imaging was used to highlight the overall morphology and detect any phase separation within the compounds, while backscattered electron imaging and EDS mapping of Calcium (Ca) was utilized to observe the distribution of the filler CaCO_3_. SEM images of the NR and ENR compounds are presented in Figure 6A,B, respectively. NR compounds, under secondary electron imaging, revealed a fairly homogeneous morphology at low magnification (micrographs on the left of Figure 6A). However, at higher magnifications, phase separation was evident in the NR-GT sample, consistent with BDS analysis, showing droplets and voids within the matrix (Figure S2A). In contrast, NR-R and NR-TOTM exhibited good homogeneity even at high magnifications. Beyond phase separation, backscattered electron images and EDS mapping revealed that GT and TOTM improved the distribution of CaCO_3_ filler compared to NR-R, likely due to the lower matrix viscosity during processing and vulcanization, which allowed the CaCO_3_ particles to disperse more homogeneously within the NR matrix. In the case of the ENR matrix, both low- (micrographs on the left of Figure 6B) and high-magnification (Figure S2B) SEM images revealed no evidence of phase separation, aligning with the findings from BDS analysis. This confirms the good compatibility of both GT and TOTM with ENR which, due to its higher polarity compared to NR, facilitate better miscibility with the two plasticizers. Backscattered electron images and EDS mapping (Figure 6B) indicated that the presence of GT does not noticeably impact the distribution of CaCO_3_ compared to ENR-R, whereas TOTM showed a less uniform filler distribution.

**Figure 6:**
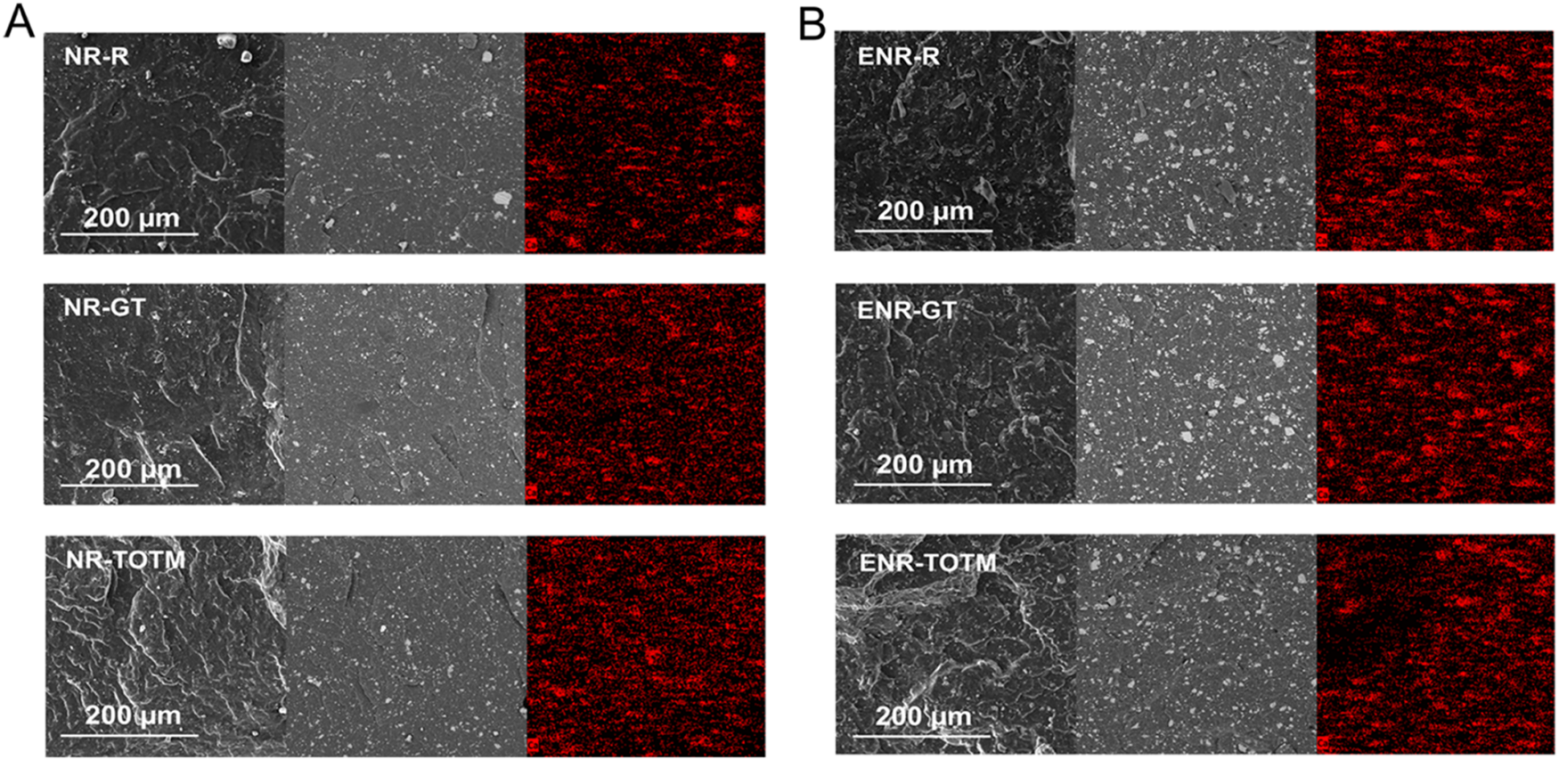
Cross-section SEM micrographs of (A) NR compounds and (B) ENR compounds. For each figure the left micrographs were captured using secondary electrons, the middle ones with backscattered electrons, and the right micrographs show the EDS mapping of calcium (highlighted in red).

## Conclusions

This study demonstrates the comparative performance of glycerol trilevulinate (GT), a biobased plasticizer, and tris­(2-ethylhexyl) trimellitate (TOTM), a petroleum-derived plasticizer, in natural rubber (NR) and epoxidized natural rubber (ENR) compounds. GT was found to be highly effective in promoting faster vulcanization and reducing the risk of reversion during prolonged exposure to heat or mechanical stress, as indicated by the significant decrease in *R*_300_ values. In contrast, TOTM tended to slow the vulcanization process and showed higher reversion under the same conditions. Structurally, both plasticizers enhanced the flexibility of the rubber network. GT promoted the formation of longer sulfur bridges, resulting in a less densely cross-linked network, which was particularly beneficial for strain-induced crystallization (SIC) in NR-based compounds. This positive effect on curing kinetics and the formation of longer sulfur bonds was likely due to GT’s ability to modify the interactions and solubility between the curing additives, facilitating more efficient cross-linking. Meanwhile, TOTM facilitated the formation of shorter sulfur bonds, leading to a more rigid cross-linked network. The dynamics of the rubber chains were also influenced by the plasticizers. GT was fully miscible with ENR, thanks to the similar polar nature of both materials, resulting in improved segmental mobility. However, in NR compounds, partial miscibility of GT led to more restricted dynamics, despite its plasticizing effect. Morphological analysis aligns well with these findings, confirming the distinct effects of the plasticizers on the cross-linking structure, material flexibility and filler distribution. Overall, GT proved to be a promising biobased alternative to traditional petroleum-derived plasticizers, particularly in applications where faster vulcanization and reduced reversion are desired. Future work could explore optimizing GT miscibility with nonpolar matrices and investigate its interactions with commonly used reinforcing fillers, such as carbon black and biobased fillers. This studycould expand GT potentiality across various rubber systems, contributing to the development of sustainable, high-performance materials in the rubber industry.

## Supplementary Material



## Notes

Mixing protocol for NR and ENR compounds (Table S1); curing parameters and cross-link density of NR and ENR formulations (Tables S2 and S3); thermal properties (*T*_d_ and *T*_g_) of NR and ENR compounds (Table S4); TGA curves for NR and ENR compounds (Figure S1); tensile test results for NR and ENR formulations (Tables S5 and S6); SEM images of NR and ENR formulations (Figure S2) (PDF)
