# Supplementary material for: Competing Effects of Plasticization and Miscibility on the Structure and Dynamics of Natural Rubber: A Comparative Study on Bio and Commercial Plasticizers
Source: ACS Polym Au. 2025 Apr 24;5(3):298–310. doi: 10.1021/acspolymersau.5c00009 (PMC12163945; doi:10.1021/acspolymersau.5c00009)
Supplement: Supplementary file 1 [file lg5c00009_si_001.pdf]

SUPPORTING INFORMATION

Competing Effects of Plasticization and Miscibility on the Structure and Dynamics of Natural Rubber: A Comparative Study on Bio and Commercial Plasticizers

Luca Lenzi [a,b], Itziar Mas-Giner [c], Micaela Degli Esposti [a,b], Davide Morselli [a,b*,] Marianella Hernández Santana [c,*], Paola Fabbri [a,b]

[a] *Department of Civil, Chemical, Environmental and Materials Engineering (DICAM), University of Bologna, Via Terracini 28, 40131 Bologna, Italy*

[b] *National Interuniversity Consortium of Materials Science and Technology (INSTM), 50121 Firenze, Italy*

[c] *Institute of Polymer Science and Technology (ICTP), CSIC, Juan de La Cierva 3, 28006 Madrid, Spain*

Corresponding authors: marherna@ictp.csic.es; davide.morselli6@unibo.it

Table S1. Mixing protocol used for producing NR and ENR compounds.

Step of compounding		Time (min)
1	Add NR/ENR	0
2	mastication	12
3	Add ZnO	15
4	Add SA	18
5	Add $CaCO_3$	21
6	Add GT/TOTM	26
7	Add CBS	29
8	Add S	32

Table S2. Curing parameters and crosslink density of NR compounds.

**Sample**	**t_{s2} (min)**	**t_{90} (min)**	**M_L (dN·m)**	**M_H (dN·m)**	**ΔM (dN·m)**	**$\rho_{crosslink} \cdot 10^{-5}$ (mol·cm^{-3})**	**R_{300} (%)**
NR-R	1.51	3.14	0.01	8.95	8.94	6.39 ± 0.10	9.05
NR-GT	0.97	2.54	0.05	6.61	6.56	4.74 ± 0.14	4.08
NR-TOTM	1.57	2.87	0.00	7.97	7.97	6.11 ± 0.04	10.54

Table S3. Curing parameters and crosslink density of ENR compounds.

**Sample**	**t_{s2} (min)**	**t_{90} (min)**	**M_L (dN·m)**	**M_H (dN·m)**	**ΔM (dN·m)**	**$\rho_{crosslink} \cdot 10^{-5}$ (mol·cm^{-3})**	**R_{300} (%)**
ENR-R	2.75	7.61	0.01	5.93	5.92	4.68 ± 0.34	3.54
ENR-GT	2.45	6.4	0.01	4.64	4.63	3.50 ± 0.10	3.02
ENR-TOTM	3.76	8.21	0.01	4.29	4.29	4.65 ± 0.32	3.26

Table S4. Thermal properties of NR and ENR compounds plasticized with GT and TOTM. Onset degradation temperatures ($T_{d,TGA}$) were determined from TGA analyses, and glass transition temperatures ($T_{g,DSC}$) were extrapolated from DSC thermograms.

**Sample**	**$T_{d,TGA}$ (°C)**	**$T_{g,DSC}$ (°C)**
NR-R	262	-55
NR-GT	226	-59
NR-TOTM	244	-59
ENR-R	300	-14
ENR-GT	221	-17
ENR-TOTM	253	-17

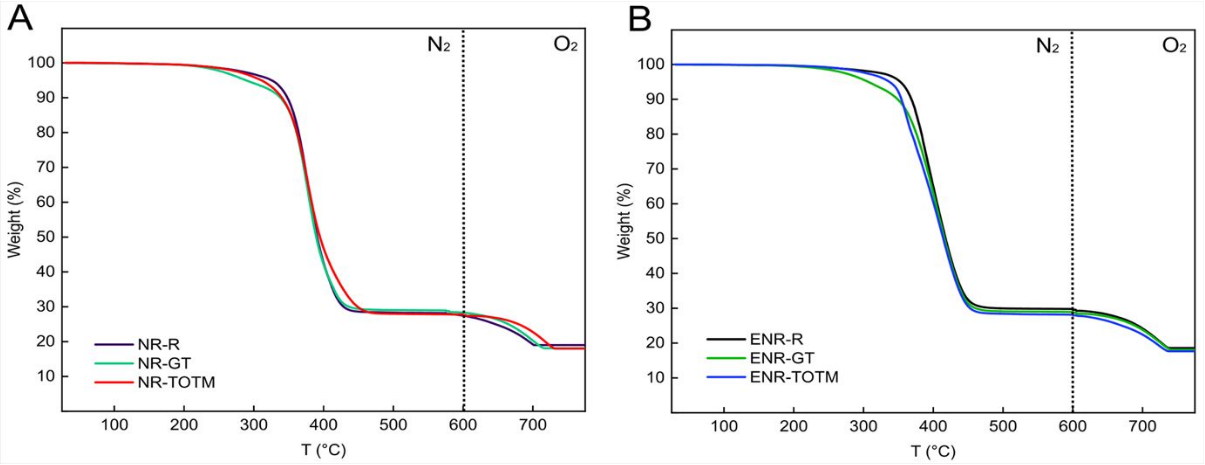

Figure S1. TGA curves of (A) neat and plasticized NR compounds and (B) neat and plasticized ENR compounds, respectively.

Table S5. Tensile test results for neat NR and its compounds with GT and TOTM plasticizers as mean ± standard deviation.

**Sample**	**M_{100} (MPa)**	**M_{300} (MPa)**	**M_{500} (MPa)**	**σ_{break} (MPa)**	**ε_{break} (%)**
NR-R	0.79 ± 0.02	1.80 ± 0.08	3.16 ± 0.26	5.85 ± 0.85	721 ± 94
NR-GT	0.52 ± 0.03	1.26 ± 0.07	2.27 ± 0.24	5.26 ± 0.46	759 ± 75
NR-TOTM	0.56 ± 0.04	1.39 ± 0.11	2.32 ± 0.17	3.90 ± 0.58	704 ± 74

Table S6. Tensile test results for neat ENR and its compounds with GT and TOTM plasticizers as mean ± standard deviation.

Sample	M_{100} (MPa)	M_{300} (MPa)	M_{500} (MPa)	σ_{break} (MPa)	ε_{break} (%)
ENR-R	0.49 ± 0.01	1.05 ± 0.03	1.66 ± 0.09	2.47 ± 0.49	669 ± 108
ENR-GT	0.36 ± 0.02	0.87 ± 0.02	1.43 ± 0.09	1.77 ± 0.11	597 ± 58
ENR-TOTM	0.38 ± 0.02	0.93 ± 0.06	1.55 ± 0.13	1.92 ± 0.16	605 ± 51

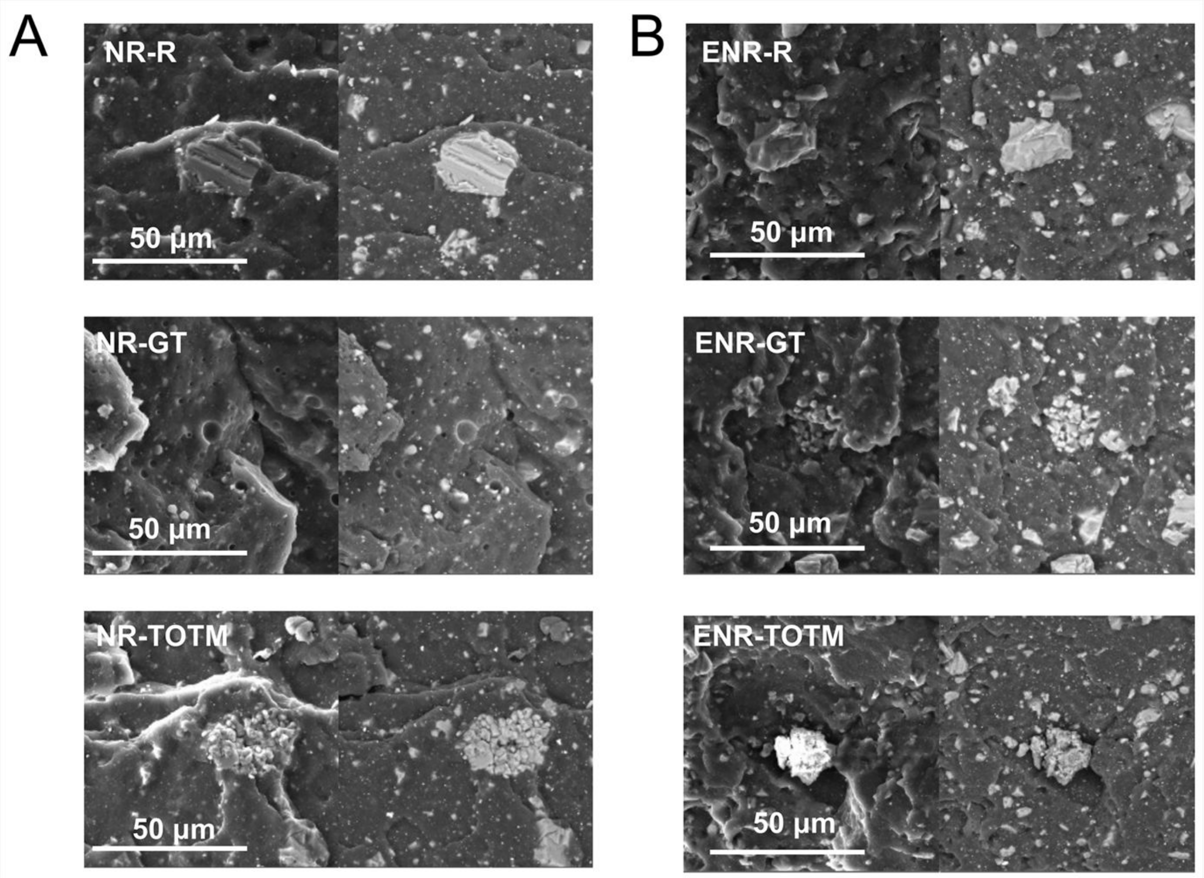

Figure S2. SEM micrographs of (A) NR and (B) ENR compounds. For each figure, the left images were captured using secondary electrons and the right ones with back-scattered electrons.
